# Effects of various hyperopia intervention levels on male college students’ gait kinematics

**DOI:** 10.3389/fphys.2023.1161711

**Published:** 2023-06-06

**Authors:** Zhaohong Zeng, Aochuan Xue, Huihui Wang, Xianjun Zha, Zhongqiu Ji

**Affiliations:** ^1^ School of Physical Education and Health, Zunyi Medical University, Zunyi, Guizhou, China; ^2^ College of Physical Education and Health, East China Normal University, Shanghai, China; ^3^ School of Physical Education and Sports, Beijing Normal University, Beijing, China

**Keywords:** hyperopia, gait, kinematics, center of gravity, joint angle, gait cycle

## Abstract

**Background:** Hyperopia is a common blurred vision phenomenon that affects postural control in gait; however, current research has focused on the alteration and correction of hyperopia’s physiological characteristics, ignoring the effect of hyperopia on gait kinematic characteristics. The effect of hyperopia on the basic form of movement walking is a worthy concern.

**Objective:** To investigate the gait kinematic characteristics of male college students with varying degrees of visual acuity (normal vision, hyperopia 150°, and hyperopia 450°), as well as to provide a theoretical foundation for the effect of visual acuity on gait and fall risk reduction.

**Methods:** Twenty-two male college students with normal visual acuity were chosen. Their vision was tested using a standard visual acuity logarithm table at normal and with 150° and 450° concave lenses. Gait kinematic data were collected under normal vision and hyperopic conditions using the PN3 Pro advanced inertial motion capture system and Axis Studio application program.

**Results and conclusion:** 1. The change of center of gravity in Pre-double support was smaller than normal vision; Late-single support and Late-swing was larger than normal vision; 2. The percentage of the double-leg support decreased; the percentage of the single-leg support and the Late-swing increased; 3. For the joints’ range of motion, Trunk flexion and extension range of motion in Pre-single support, Late-double support and Pre-swing smaller than normal visual acuity, and Late-swing larger than normal; hip internal abduction and adduction and internal and external rotation are larger than normal vision in Late-single support; knee and ankle in abduction and adduction direction are larger than normal vision in the swing stage; hip flexion and extension, internal external rotation are larger than normal vision in the swing stage. Hyperopic interventions have an impact on the kinematic characteristics of gait in male college students, mainly in terms of altered balance, increased instability, increased difficulty in maintaining trunk stability, and increased risk of injury.

## 1 Introduction

Walking is a fundamental mode of locomotion in humans, a physiological activity controlled by the nervous system and involving the coordinated action of muscles, bones, and joints throughout the body (Morelli and Morelli, 2021; Chen et al., 2022). Vision, vestibular, and proprioception are all important factors in gait, and vision, as the primary sensory input, can accurately respond to the body’s relative position to the external environment, regulate gait postural control, and prevent falls (Morelli and Morelli, 2021). Postural reflexes and anticipatory postural regulation are reduced when there is visual impairment, affecting the body’s postural control. Hyperopia is a common visual abnormality that affects the input of visual information in gait. However, the hyperopia people will only wear glasses in the near vision situation such as reading books and newspapers, but the abnormal posture control caused by hyperopia exists all the time, so the posture control during the gait of the hyperopia people is worth studying (Chinese Journal of Rehabilitation Medicine, 1993).

In the study of gait in sports biomechanics, kinematic data are often collected using kinematic data acquisition by infrared capture systems and inertial motion capture systems, kinetics are analyzed using dynamometers and plantar pressure, and muscle force is analyzed using electromyography (Brodoehl et al., 2015; Wiesinger et al., 2022). Based on the fact that changes in kinetic and EMG-related data are important causes of alterations in gait kinematic characteristics, the external appearance of postural control imbalances can be found in kinematic analysis. Kinematic analysis is a scientific method to study the changes in the temporal and spatial movement patterns of the limbs during walking. The kinematic changes can be reacted to the center of gravity (Lehman et al., 2008), the percentage of gait cycle (Yang and Chen, 2019; Ma et al., 2021) and joint motion angles (You et al., 2003; Dai and Li, 2016; Matchar et al., 2017; Wang et al., 2018; Liu et al., 2022). The more stable the center of gravity and the relatively more the proportion of double support, the better the postural control.

Visual impairment (Morelli and Morelli, 2021) and visual fatigue (Davids, 2014) have been evidenced to affect balance and produce falls, but there is insufficient evidence to demonstrate the effect of vision, especially hyperopia, on postural control in gait. The purpose of this study was to investigate the effect that different levels of hyperopia would have on the kinematic characteristics of gait. The hypothesis of this study is that changes in center of gravity, the percentage of gait cycle and joint motion angles will take effect after a hyperopic intervention.

## 2 Subjects and methods

### 2.1 Designs

Experiment datas on gait kinematics for male college students were used repeated measures of one-way ANOVA under normal vision and hyperopia conditions of 150° and 450°.

### 2.2 Time and location

The experiment will last 20 days and begin in July 2022. The test will take place at Zunyi Medical University’s Experimental Center of Sports Rehabilitation.

### 2.3 Participants

Thirty male college students with normal vision from Zunyi Medical University were recruited. After eligibility assessment, a total of 22 participants met the experimental requirements, the participants’ average age is 20.82 ± 1.40 years, their average height is 174.86 ± 3.27 cm, their average weight is 65.48 ± 9.48 kg, and their average BMI is 21.32 ± 2.54 kg/m^2^. Before the trial, all participants provided written informed consent and understood the experimental process and purpose. This study was reviewed and approved by the Ethics Committee of the Zunyi Medicai University.

Criteria for Inclusion: 1. Normal vision in both eyes (visual acuity of both naked eyes 5.0); 2. No recent history of eye or vision correction surgery; and 3. No impairment of walking or motor function.

Criteria for Exclusion: 1. Exclusion Poor visual acuity, defined as visual acuity of less than 5.0 in either eye; 2. The presence of motor impairment; 3. The inability to finish the exam on one’s own exclusion factors.

### 2.4 Methods

#### 2.4.1 Data collection for visual acuity

The visual acuity of the participants is collected by professional testers when they are in normal vision and wearing 150° and 450° concave lenses. The environment of the place is required to be clean, neat and quiet; the size of the place and the light meet the standard, the distance is up to the standard, the illumination is uniform, constant, non-reflective and non-dazzling, and direct sunlight is prohibited. The visual acuity test was conducted using a standard logarithmic visual acuity table, which consisted of 12 rows of “E" with different sizes and openings in different directions, and measured visual acuity in the range of 4.0–5.2; each row was labeled with a number, the visual acuity table was hung at a height such that most of the measures had a horizontal line of sight at the position of 5.0 rows of visual. The participants were in a quiet state 10 minutes before the test and could not perform a strenuous exercise; during the test, the participants were kept upright and was randomly selected normal vision, 150° or 450° hyperopic intervention by the tester to test the visual acuity. The right eye test is as follows: the participants pointed out the direction of each viewpoint in turn, starting from the largest viewpoint. The examination was completed when the participant made one error in each row from 4.0 to 4.5, or two errors in each row from 4.6 to 5.0, or three errors in each row from 5.1 to 5.3. The visual acuity represented by the previous row is the result of the participant’s visual acuity test. The results were recorded according to the five-point recording method. To guarantee the accuracy of the data, 5% of the participant were chosen for evaluation after the visual acuity test.

Grading of visual acuity (Tong et al., 2000; Vivekanand et al., 2021): Mild hyperopia was defined as 300° of hyperopia, moderate hyperopia as 300°–600° of hyperopia and Severe hyperopia as >600° of hyperopia; intermediate values of 150° and 450° of low and moderate hyperopia, respectively, were chosen. To simulate hyperopia, 150° and 450° concave lens glasses were used: 150° concave lens glasses were used for mild hyperopia, and 450° concave lens glasses were used for moderate hyperopia. The test was not performed because high hyperopia is very blurry.

#### 2.4.2 Data collection for gait

##### 2.4.2.1 Experimental equipment

**FIGURE 1:**
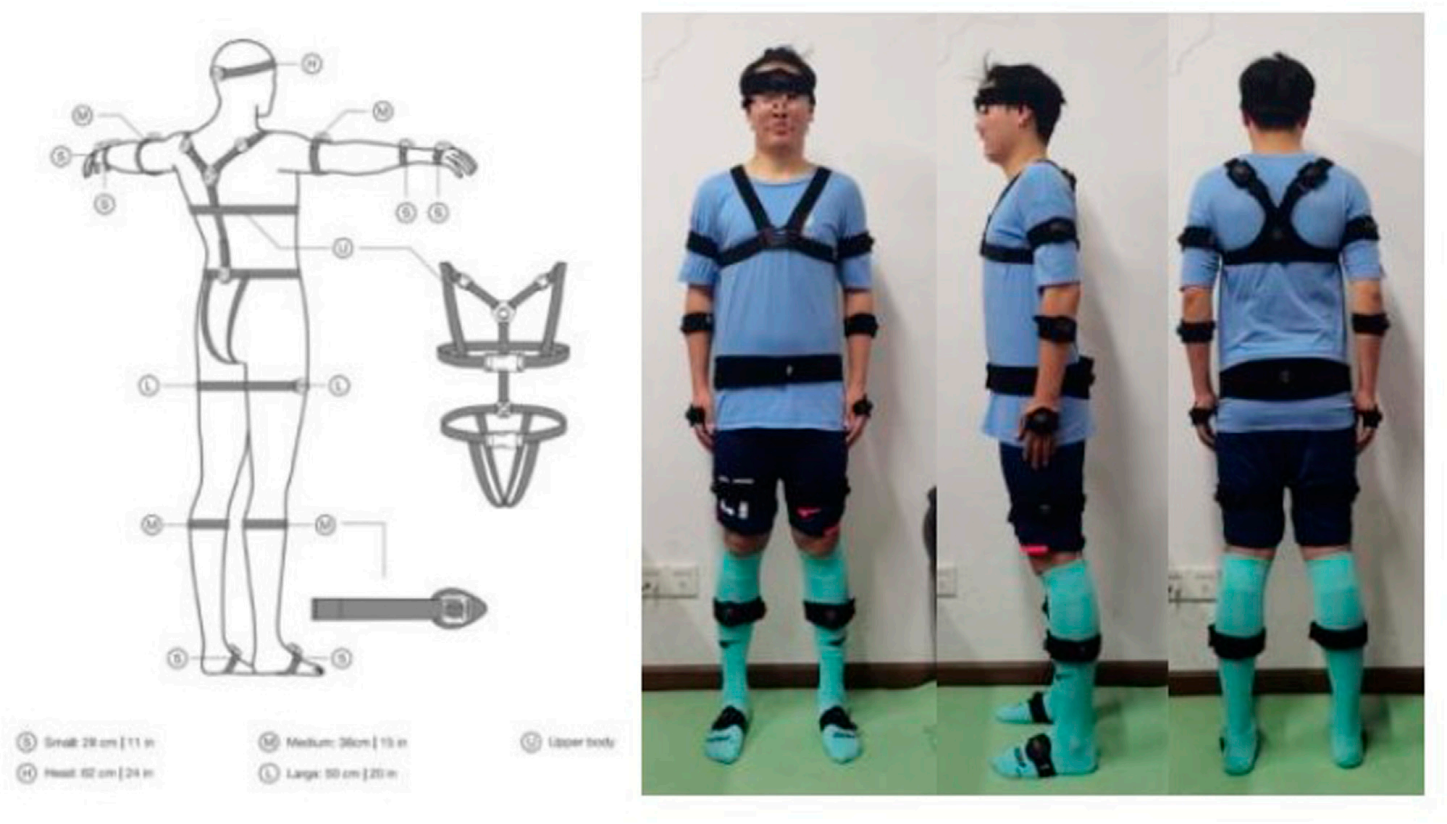
Wearing sensors.

**FIGURE 2:**
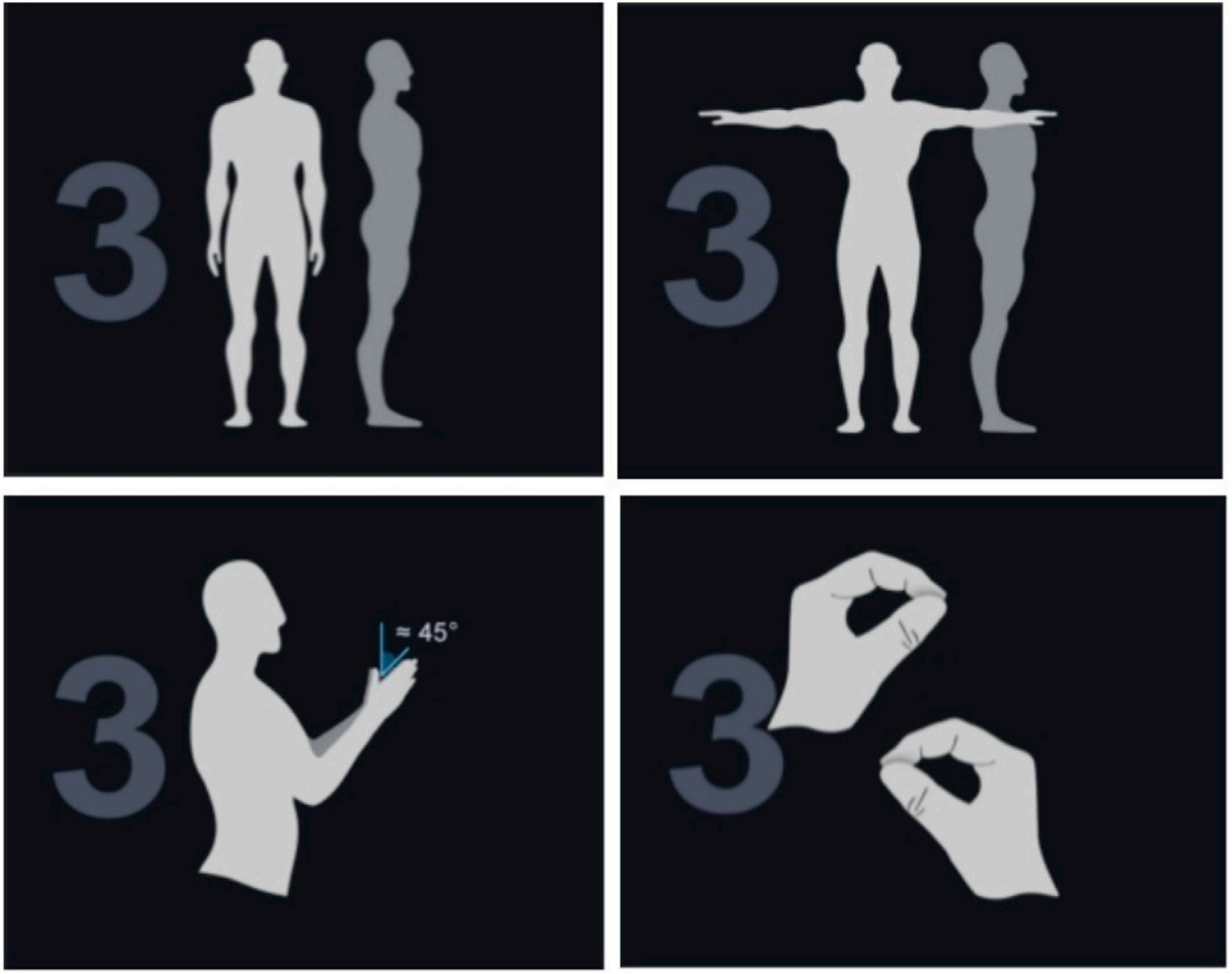
A-T-B-P posture.

The Perception Neuron 3 Pro advanced inertial motion capture system was used to collect gait kinematic data for this study. Morphological data such as head length, neck length, torso length, shoulder width, femur width, upper arm length, forearm length, hand length, thigh length, calf length, foot length, heel height, arm span, and so on were measured and recorded by professional testers and imported into the skeletal size database to create a personalized model of the participant. Wearing sensors: 16 sensors were placed at the midpoint of the forehead, the upper third of the scapulae, the forearm, the lateral midpoint of the thigh, anterior side of calf, the fourth lumbar vertebra, the dorsum of the foot and the dorsum of the hand (as shown in Figure 1). After the participant has finished wearing the device, the software is opened for warm-up, the sensors are connected, and the participant performs posture calibration using the A-T-B-P action (as shown in Figure 2): A-posture is when the body is upright, the arms are down, and the palms of the hands face the body. Adjust the space between your feet to be the same width as your hips, with both feet facing forward. The T-pose is based on the A-pose, with the arms raised sideways to parallel to the horizontal, while the palms face down. The B-Posture is based on the A-Posture with the shoulders slightly extended forward and the elbows flexed at 90°, while the palms are placed against each other in the middle of the chest; and the elbows, wrists and middle fingers are in a line, while the thumbs and other four fingers are at an angle of about 45°. P-position is to straighten the thumb and gently pinch the index finger, while requiring the thumb to be located in a straight line from the heel to the tip as much as possible, with the index finger naturally bent to touch the tip of the thumb and the remaining three fingers naturally bent. When the calibration is finished, a personalized model is created and ready for testing.

##### 2.4.2.2 Gait data collection process

Participants wore 150°, 450° concave lenses, or normal without glasses and walked barefoot independently in a sensor sensing area of 10m*3m, with the requirements of the test being uniform speed and smooth walking. If there is external interference, a sensor signal that is outside the test range, or an abnormal sensor signal during the experiment, the test is considered a failure and must be re-tested. Three times successful gait kinematic data collections for normal vision and hyperopia 150° and 450° vision were completed.

##### 2.4.2.3 Data processing

The moment of landing and the moment of leaving the ground is the key point to divide the gait cycle, the gait cycle can be defined as one side of the feet following the ground to the side of the heel landing again, according to the key point, we transfer the sensor data to Axis Studio software, wthere the test video is simulated at the 3D level based on kinematic data and divided a gait cycle into: Pre-double support, Pre-single support, Late-single support, Late-double support, Pre-swing and Late-swing. Pre-double support: left heel pointing-right toe off the ground; Pre-single support: right toe off the ground - right heel passing the left foot; Late-single support: right heel passing the left foot-right heel landing; Late-double support: right heel landing-left toe off the ground; Pre-swing: Left toe off the ground-Left heel passing right heel. Late-swing: Left heel passing right heel-Left heel pointing to the ground. (Yang and Chen, 2019).

The following are the main collection indicators: the range of vertical center of gravity change in each phase of the gait cycle, percentage of each phase of the gait cycle, the joint motion angle of the trunk and the lower limbs of the hip, knee and ankle around the frontal, sagittal and vertical axes.

### 2.5 Statistical analysis

Statistical analysis of indicators was performed using SPSS 24.0 software, and the data were described by mean ± standard deviation. One-way ANOVA with repeated measures was used for comparison between groups, and LSD multiple comparisons were used for *post-hoc* tests, with the significance level taken as α = 0.05.

## 3 Results

### 3.1 Visual acuity test results

According to Table 1, Normal vision tests for male university students, all with visual acuity values above 5.0, with a 150° concave lens: left eye vision is 5.11–5.19, right eye vision is 5.10–5.20 and binocular vision is 5.17–5.22; with a 450° concave lens: left eye vision is 4.58–4.91, right eye vision is 4.57–4.89 and binocular vision is 4.52–4.84.

**TABLE 1:** The basic information of participants’ visual

		Visual acuity	Lower limit	Upper limit
Vision in the left eye	Normal	5.13±0.09	5.09	5.17
	hyperopia 150°	5.15±0.09	5.11	5.19
	hyperopia 450°	4.75±0.38	4.58	4.91
Vision in the right eye	Normal	5.15±0.10	5.11	5.19
	hyperopia 150°	5.15±0.11	5.10	5.20
	hyperopia 450°	4.73±0.37	4.57	4.89
Binocular vision	Normal	5.22±0.08	5.18	5.25
	hyperopia 150°	5.20±0.07	5.17	5.22
	hyperopia 450°	4.68±0.36	4.52	4.84

Table Note: Hyperopia 150° is the visual acuity measured by wearing a 150° concave lens, and hyperopia 450° is the visual acuity measured by wearing a 450° concave lens.

### 3.2 Change of center of gravity

**FIGURE 3:**
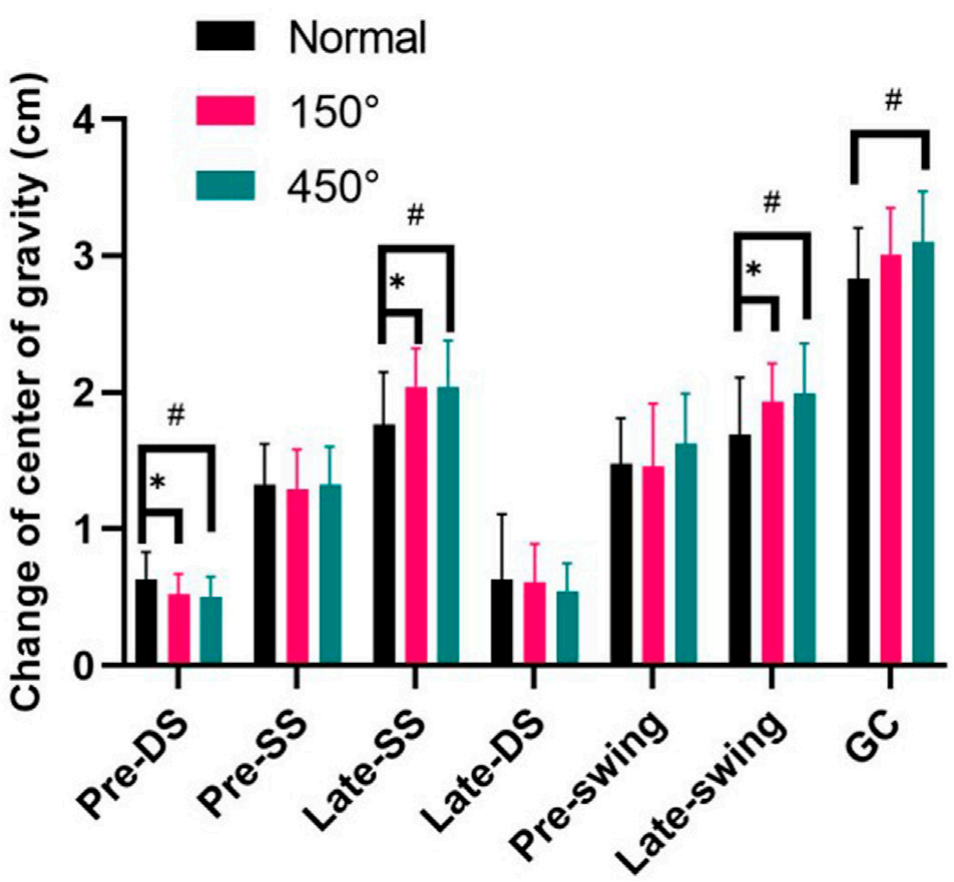
Change of center of gravity.

Figure 3 demonstrates that for male college students with hyperopia of 150° and 450°, the range of change in center of gravity in Pre-double support was significantly smaller than normal vision (*p* < 0.05); the range of change in center of gravity in Late-single support and Late-swing was significantly larger than normal vision (*p* < 0.05); and only the hyperopia of 450°, all gait cycle’s time lager than normal visual (*p* < 0.05).

### 3.3 Percentage of gait cycle

**FIGURE 4:**
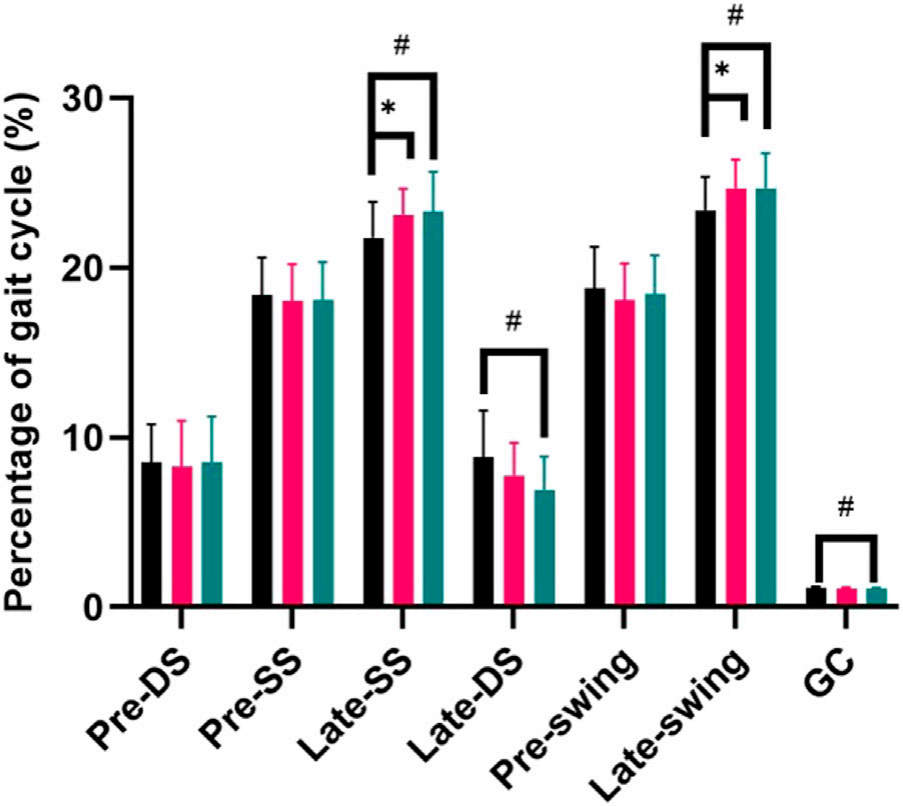
Percentage of giat cycle.

As shown in Figure 4, in the hyperopic 150° and 450° conditions the percentage of the gait cycle in Late-single support and Late-swing was significantly larger than normal vision (*p* < 0.05); in the hyperopic 450° condition, the percentage of the gait cycle in Late-double support and the all gait cycle’s time was significantly less than normal vision (*p* < 0.05).

### 3.4 The trunk’s range of motion

**FIGURE 5:**
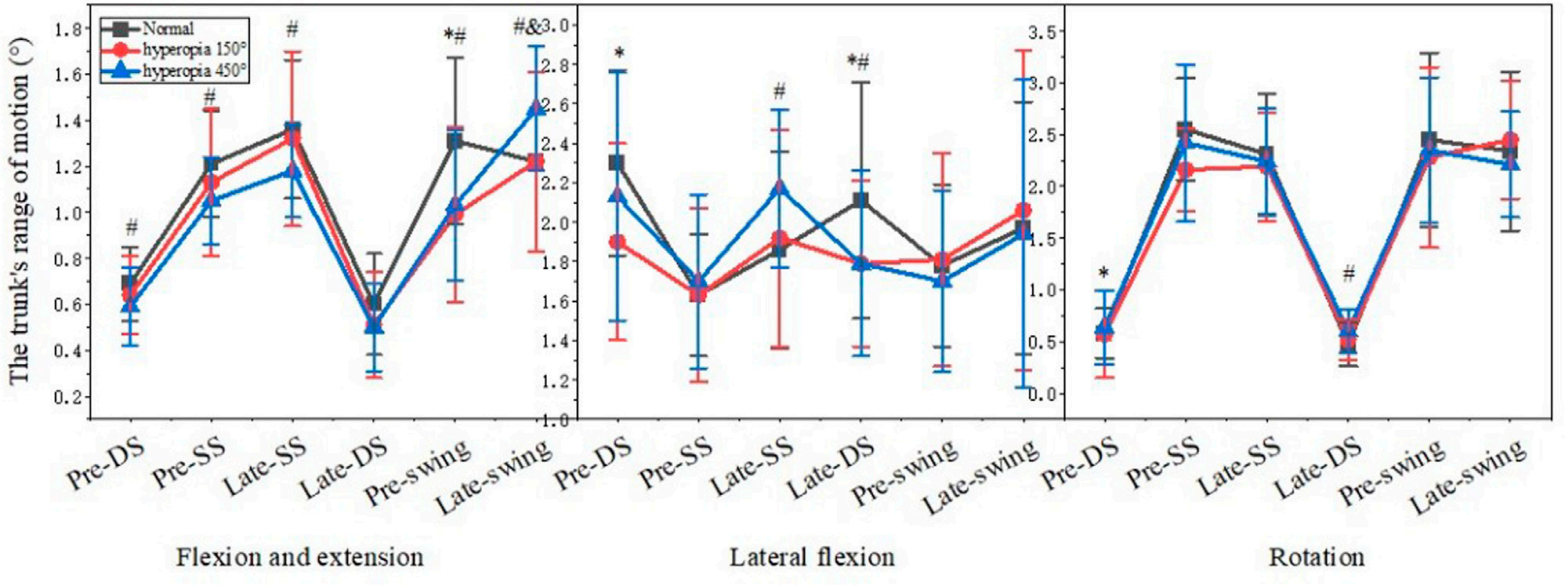
The trunk’s range of motion.

Figure 5 shows that in the 150° and 450° hyperopia in the Late-double support of the trunk lateral flexion direction, in the Pre-swing of the trunk flexion and extension direction for male college students were significantly smaller than normal visual acuity (*p* < 0.05). In the 150° hyperopia, in Pre-double support the trunk joint movement of the lateral flexion and rotation were significantly smaller than normal visual acuity (*p* < 0.05), and in the 450° hyperopia, in Pre-double support, Pre-single support and Late-single support, the trunk joint movement of the flexion and extension were significantly smaller than normal visual acuity (*p* < 0.05).

### 3.5 The lower limb joints range of motion

**FIGURE 6:**
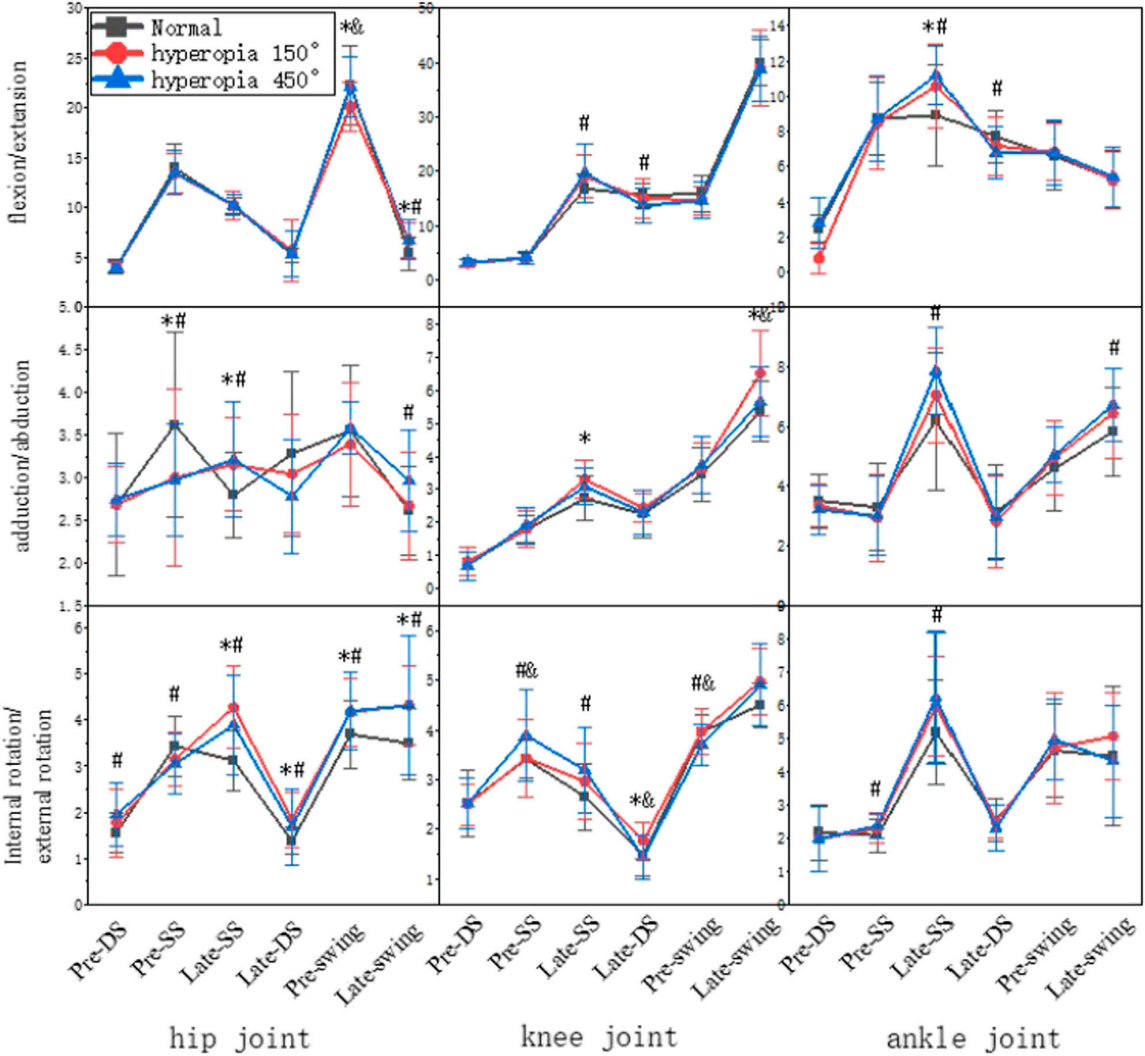
The lower limb joint’s range of motion.

The range of variation was larger in the Late-single support and Late-swing, as shown in Figure 6. In 150° and 450° hyperopia, in the Pre-single support the hip joint motion of the internal abduction and adduction were significantly smaller than normal visual acuity in the Late-single support the hip joint motion of the internal abduction and adduction, internal rotation and external rotation, and ankle joint motion of the flexion and extension were significantly larger than normal visual acuity; in the Pre-swing the hip joint motion of the internal rotation and external rotation and in the Late-swing the hip joint motion of the internal rotation and external rotation and flexion and extension were significantly larger than normal visual acuity (*p* < 0.05). In 150° hyperopia the knee joint motion of the internal abduction and adduction in the Late-double support was significantly larger than normal visual acuity; and the knee joint motion of the internal abduction and adduction was significantly larger than normal visual acuity in the Late-swing and Late-single support (*p* < 0.05). In 450° hyperopia, in the Pre-swing the knee motion of the internal rotation and external rotation was significant differences with normal visual acuity; in the Late-swing the ankle joint motion of the internal abduction-adduction, was significantly larger than normal visual acuity (*p* < 0.05).

## 4 Discussion

Hyperopia is a condition in which the eye at rest forms a focal point on the back of the retina of parallel light. It is caused by a physiological or pathological shortening of the anterior posterior axis of the eye, a reduction in the surface curvature of the refractive body, and a weakening of the lens’s refractive power. Light pollution is becoming more serious as society develops, people’s vision is gradually deteriorating, and hyperopia is also trending towards youth. Hyperopia affects postural control and muscle preactivation of the body’s movement between travels; for example, human gait balance is primarily dependent on visual regulation. In our study, using 150° and 450° concave lens glasses, investigated the effects of different degrees of hyperopia on the kinematic characteristics of male college students’ gait. During the visual acuity test, the 450° hyperopia results were significantly smaller than normal visual acuity and 150° hyperopia, and there was no significant difference between the normal visual acuity and in the 150° hyperopia, but the participants’ gait kinematic characteristics changed at 150° hyperopia, which may be due to the fact that the participants wore 150° concave lenses to test their visual acuity by squinting, they adapted to the change in visual acuity, and the visual acuity test is a relatively static process, whereas walking is a dynamic process that is participant to changes in the surrounding environment and requires more adjustment, so the visual acuity test results did not differ significantly from normal visual acuity at 150° of hyperopia, but the kinematic characteristics of the gait cycle did.

The vertical center of gravity trajectory of the body shows a sinusoidal trajectory motion of about 5°, while the pelvic trajectory is a forward and backward rotational motion of about 8° during the gait cycle. Non-physiological changes in the center of gravity’s trajectory may indicate an increased risk of movement (Bolink et al., 2012; Eckel et al., 2012). For example, a small range of body center of gravity changes makes the body more stable and less likely to fall when walking, whereas an increased range of center of gravity changes may impair balance function (Lefeber et al., 2020; Moon et al., 2022). The results showed that the change in center of gravity during the Late-double support in male college students with hyperopia vision was smaller than normal vision, which may be due to the abnormal visual input under the hyperopia condition, which cannot greater identify the spatial position of the body and relies on reducing the change in center of gravity to maintain body stability; however, the range of change in center of gravity during the single-leg support was larger than normal vision. Because the body is unable to regulate the change in center of gravity, there is a risk of falling (Zhao and Zhou, 2003; Liu et al., 2022). Therefore, balance training should be increased for the hyperopia, especially the elderly, to increase the stability of the hyperopia elderly during walking and reduce the occurrence of falls.

The stability of the human body during the double-support period is larger than that of the single-leg support throughout the gait cycle (Duclos et al., 2012). In double-leg support, both feet are on the ground, the center of gravity is offset to the both feet, and the body weight is borne by both feet; in single-support, one foot is off the ground, the center of gravity is offset to the supporting one leg, and the body weight is borne by one lower limb; and the support area of the center of gravity is reduced compared to that of double-support, so the more the proportion of the double-support, the more stable the body (Liu et al., 2022). In our study, in the hyperopic condition the percentage of the double-leg support decreased; the percentage the single-leg support and the Late-swing increased, which are consistent with a previous study, which found that the proportion of single-leg support time in obese children was higher than in normal-weight children (D'Hondt et al., 2011). Therefore, it can be inferred that hyperopia leads to a certain degree of change in the percentage of gait cycle, resulting in an increase in instability time and a decrease in stability time, which affects the stability of the whole gait cycle. Therefore, stability training for hyperopic people is also very important, especially in the single-leg support state.

The trunk plays a pivotal role in gait, and its non-physiological forward flexion and extension as well as lateral flexion and rotation can cause movement tension and affect walking stability; trunk posture control is closely related to the angle of lower limb joint movements, and changes in trunk posture cause changes in the angle of lower limb joint movements, and lower limb joint movements also regulate changes in trunk posture (Lang and Ji, 2003). Studies have shown that anterior trunk flexion causes forward head flexion and tends to cause anterior pelvic movement, limiting forward and backward hip rotation around the vertical axis and reducing stride length; posterior trunk extension tilts the head back, leading to a longer support phase (Haruyama et al., 2019). In addition, the backward tilt angle of the trunk is too large to cause the body to form an anti-support state; the forward pitch angle is too large to limit the flexibility of the hip joint; the left and right swing amplitude is large to generate excess horizontal component forces, which tend to affect the ability of visual and vestibular sensory regulation of posture, all of which are detrimental to the stability of human walking. In this study, the trunk flexion and extension angle in the Late-swing under the hyperopic 450° condition was larger than normal, at same time, the hip inversion and abduction angle was significantly larger than normal, and this phenomenon may be due to the fact that the hip and trunk are adjacent to each other, and the increase in trunk flexion and extension angle may be related to the hip angle and the proportion of each phase in the gait cycle, and it is mainly the rectus femoris muscle in the Late-swing that makes the lower leg swing forward and maintains the stability of the lower leg in the Late-swing, and it is also one of the main force-generating muscles in hip flexion (Xiong et al., 2022). Thus, it was confirmed that trunk postural control and hip joint movement angle are closely related and that the transmission, analysis and integration of sensory information related to trunk control is reduced in participants, which affects trunk control (Petersen et al., 2022). Therefore, this study suggests that people with hyperopia should strengthen the training of core muscles to increase the postural control of the body in order to reduce the probability of falling.

Postural control is primarily regulated by sensory inputs such as visual, kinesthetic, and vestibular sensations. Changes in the structure and function of the visual system weaken visual sensory information input and make it more difficult for the body to identify spatial position and motion feedback while walking, which eventually leads to imbalance in postural control and falls, so the body will regulate by changing the relative position of joints or joint range of motion to avoid danger. In our study, the three lower limb joints are altered to varying degrees of joint range at various stages, and the stages with the most differences in joint changes are the Late-single support and Late-swing, which may be one of the reasons why most falls occur in Late-single support and Late-swing for example, studies have shown that falls in older adults occur in greater numbers in Late-single support and at the moment of the landing. Injuries in athletes occur more frequently during the swing period, and the risk of injury in both the single-leg support and the swing phase is higher than in the other phases (Duclos et al., 2012; Ji et al., 2015; Niu et al., 2020). Based on the foregoing, it can be deduced that the phases with the greatest variation in the range of motion of the participants’ joints under hyperopic conditions are the Late-single support and Late-swing, both of which are injury-prone.

Joint coordination is essential for maintaining gait stability (Ippersiel et al., 2021; Tang et al., 2022). Visual input is impaired, walking is unstable, and the body regulates mechanoreceptor input-output by inducing plastic changes in the central nervous system, strengthening information transmission pathways, enhancing proprioception, and repeatedly adjusting and controlling body center of gravity and limb position (Ribeiro and Oliveira, 2007; Tang et al., 2022). According to the findings of this study, the lower limb joint motion angle of male college students is increasing, indicating that the non-physiological increase in the lower limb motion angle of gait in the long-term hyperopia population can affect the trajectory of the center of gravity and predispose to chronic wear and tear of the lower limb joints, the increased angle of motion in the direction of knee flexion and extension increases the load on the anterior cruciate ligament, which can easily cause knee injuries. Furthermore, the rising trend of lower limb joint motion angle occurs mostly during the single support phase and swing phase, when the human body has a small support area and the range of change of center of gravity increases, increasing the risk of falling and injuries. Therefore, people with hyperopia should reduce the risk of injury through joint stability and coordination training.

### 4.1 Limitations

There are some limitations in this study: 1. Walking is a coherent movement done by the whole body in coordination, and the influence of vision on trunk, upper limb and lower limb coordination should be considered comprehensively. 2. Walking speed is not strictly defined in this experiment, which may have some influence on the experimental results. 3. For the study of center of gravity, this experiment selected the change of center of gravity in vertical direction, therefore in future studies, the change of center of gravity in up and down, left and right, and front and back should be selected for a comprehensive study. 4. In this study, we used a concave lens to intervene in a normal population, and even though we gave the participants enough adaptation time, there is still a possibility that it may not be able to fully simulate the hyperopic state, and there is a gap between it and the truly hyperopic population.

## 5 Conclusion

Hyperopic interventions have an impact on the kinematic characteristics of gait in male college students, mainly in terms of altered balance, increased instability, increased difficulty in maintaining trunk stability, and increased risk of injury. Therefore, it is recommended to increase gait training and increase postural control in walking for hyperopic older adults to reduce falls and injuries and improve quality of life in old age.

## Data Availability

The original contributions presented in the study are included in the article/Supplementary Material, further inquiries can be directed to the corresponding author.
